# Molecular reclassification reveals low prevalence of germline predisposition in children with ependymoma

**DOI:** 10.1186/s40478-023-01594-x

**Published:** 2023-06-12

**Authors:** Jon Foss-Skiftesvik, René Mathiasen, Thomas van Overeem Hansen, Karin Wadt, Kjeld Schmiegelow, Ulrik Kristoffer Stoltze

**Affiliations:** 1 Department of Neurosurgery, Rigshospitalet University Hospital, Blegdamsvej 9, Copenhagen, 2100 Denmark; 2 The Pediatric Oncology Research Laboratory, Rigshospitalet University Hospital, Copenhagen, Denmark; 3 Faculty of Health and Medical Sciences, University of Copenhagen, Copenhagen, Denmark; 4 Department of Pediatrics and Adolescent Medicine, Rigshospitalet University Hospital, Copenhagen, Denmark; 5 Department of Clinical Genetics, Rigshospitalet University Hospital, Copenhagen, Denmark

**Keywords:** Ependymoma, Genetic predisposition, Tumor methylation profiling, Germline, Sequencing

## Main text

Ependymoma is the second most common malignant childhood brain tumor, accounting for approximately 6% of all central nervous system (CNS) tumors in children [6]. Nonetheless, our knowledge of its underlying etiology is sparse. Since the introduction of next-generation sequencing, several pan-childhood cancer germline sequencing studies have been published covering more than 1,400 children with CNS tumors, including 191 ependymomas (as recently reviewed [4]). In combination, these studies report 4.7% (9/191) of children with ependymoma to harbor pathogenic germline variants likely underlying the cancer pathogenesis, although individual study estimates range from 0 to more than 20%. Moreover, differing approaches to variant classification and the lack of molecular tumor diagnostics and population-based study designs challenge drawing inferences about the true nature of genetic predisposition for these children. In 2022, we, therefore, reported the findings from our nationwide, population-based germline whole-genome sequencing study specific to children with molecularly classified ependymoma diagnosed in Denmark years 2000–2021 in Acta Neuropathologica Communications [4].

**Fig. 1:**
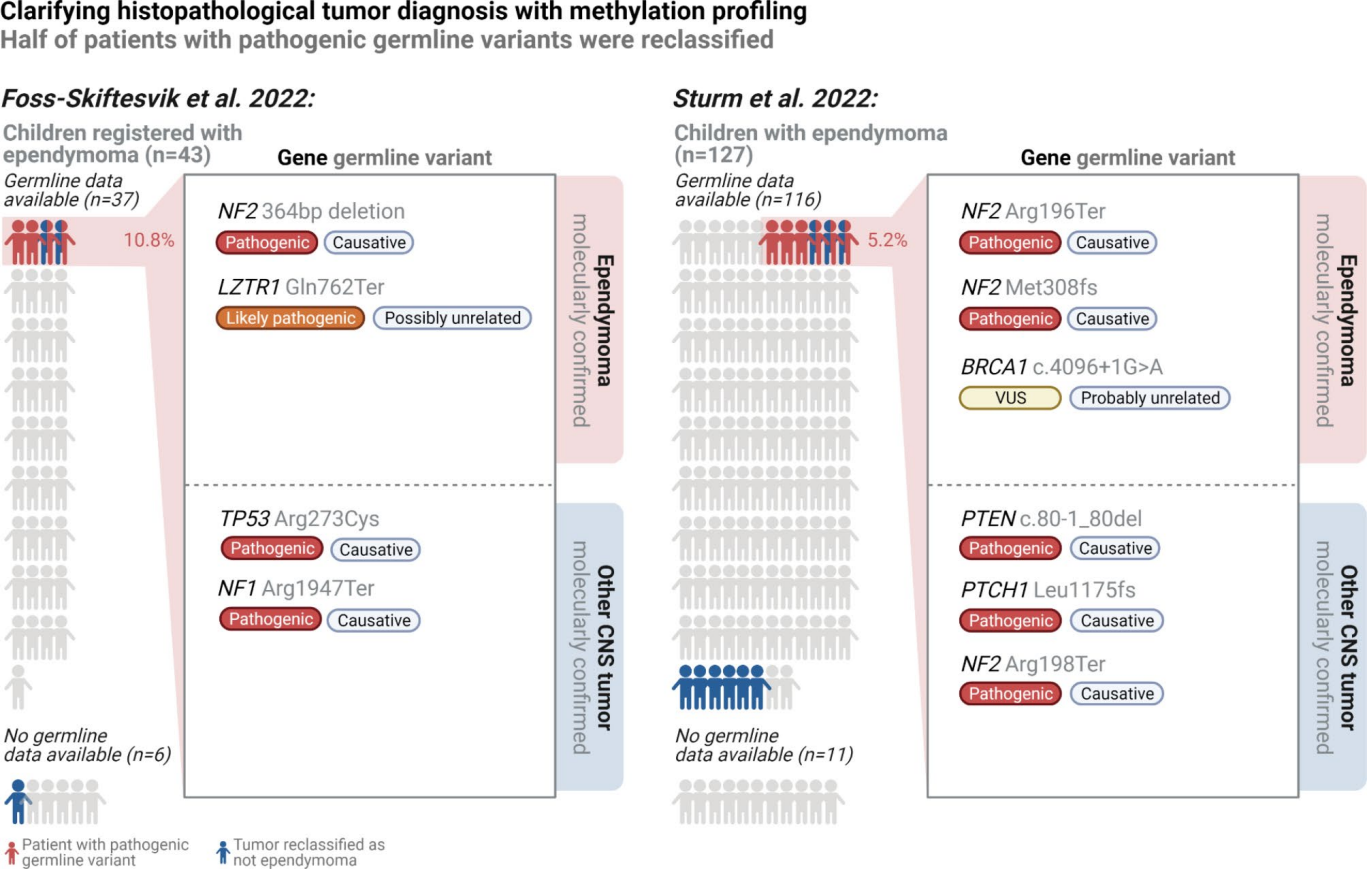
**Overview of diagnostic reclassification of original histopathological ependymoma diagnoses following tumor methylation profiling.** In Sturm et al.’s study, six additional children were reclassified from other non-ependymoma tumor types to ependymoma following tumor methylation profiling (not shown). Targeted germline sequencing was performed for five without detection of pathogenic variants. Adapted from Foss-Skiftesvik et al. Acta Neuropathologica Communications(2022) 10:123, https://doi.org/10.1186/s40478-022-01429-1 with permission under the Creative Commons Attribution 4.0 International License (http://creativecommons.org/licenses/by/4.0/). Created with BioRender.com

More recently, Sturm et al. published an international population-based prospective study of 1,204 children with CNS tumors undergoing tumor methylation profiling, targeted tumor sequencing as well as targeted germline sequencing of 47 known cancer predisposition genes [9]. This cohort included 127 children with histopathologically diagnosed ependymoma. Similarly to the 7.7% reclassification rate in our report [4], tumor methylation profiling indicated amendment to a non-ependymoma tumor type in 7.1% in Sturm et al.’s cohort. This included a histopathologically diagnosed ependymoma WHO grade 3 of the third ventricle, which was revised to an embryonal tumor with multilayered rosettes (ETMR), just as in our cohort. Furthermore, six children with other tumor types were reclassified as having ependymomas following tumor methylation profiling. Of the 116 children with histopathologically diagnosed ependymoma who underwent targeted germline sequencing in the study by Sturm et al., 5.2% (n = 6) were found to harbor pathogenic germline variants considered to be causative of disease (*NF2* in three patients, *BRCA1*, *PTEN* and *PTCH1*). However, following molecular tumor classification using the predicted methylation class with the highest calibrated score from version 12.5 of the Heidelberg classifier [2], the prevalence declined to 2.7% (3/112; *NF2* in two patients and *BRCA1*)(Fig. 1.). Our findings were very similar with 10.8% and 5.9% harboring pathogenic germline variants, respectively, of which the latter were limited to *NF2* and *LTZR1*.

In other words, 50% of children with histopathologically diagnosed ependymoma *and* identified pathogenic germline variants were reclassified to having a non-ependymoma tumor type following methylation profiling in both studies (3/6 and 2/4, respectively, Fig. 1). Thus, diagnostic reclassification from histopathological ependymoma to a molecularly classified non-ependymoma tumor type was significantly more likely in children harboring pathogenic germline variants in both Sturm et al.’s and our cohort (5/10 vs. 6/139, p-value < 0.001, Fisher’s exact test, combined data). The background for this higher rate of misclassification remains unknown and merits further investigation.

Our previous best prevalence estimate for predisposing germline variants in children with ependymoma was 3.4%. This was based on pooling our recently reported cohort with a comprehensive quantitative review of the existing literature [4], which, of note, is predominated by non-population-based studies of children with non-molecularly classified intracranial ependymoma. From the current reanalysis, it is evident that predisposing pathogenic germline variants are significantly less common in children with molecularly classified ependymoma compared to other childhood CNS tumors overall (5/146 vs. 98/922 (non-ependymoma childhood CNS tumors included in Sturm et al.’s study), p-value 0.004, Fisher’s exact test).

Interestingly, in children with ependymoma, all previously reported pathogenic germline *TP53* and *NF1* variants have either subsequently been reclassified as benign, or described in children for whom tumor methylation profiling has not been reported [1, 3, 5, 7, 8, 10, 11]. This calls into question the link between both Li-Fraumeni Syndrome and neurofibromatosis type-1 and (molecularly classified) ependymoma. Although the *BRCA1* variant reported by Sturm et al. has been interpreted by several sources as likely pathogenic (ClinVar accession number VCV000037565.30), its role in childhood ependymoma may also be questioned as the variant more recently has received class 3 classification (variant of unknown significance) following expert panel evaluation by the Evidence-based Network for the Interpretation of Germline Mutant Alleles (ENIGMA). Unfortunately, somatic loss-of-heterozygosity status for the pathogenic *LTZR1* variant from our cohort was not available, and its causal relationship with ependymoma may also be considered speculative. If excluded (the *BRCA1* and *LZTR1* variants), the prevalence of rare pathogenic germline variants in children with molecularly classified ependymoma decreases further to less than 3% (2.1%, 3/146), with mutations observed exclusively in *NF2* in children with methylation class spinal ependymoma.

These data emphasize the need for both germline and tumor DNA profiling in children with CNS tumors and highlight the exceptional scarcity of germline mutations in children with molecularly classified intracranial ependymoma. Identification of cancer predisposition syndromes other than neurofibromatosis type-2 should warrant diagnostic reconsideration in children with ependymoma for whom molecular classification has not been performed. We encourage future studies of germline predisposition in children with ependymoma to include tumor molecular classification. As we have previously shown, this is feasible also for retrospective studies using archived tumor samples stored for more than 20 years [4].

## Data Availability

Not applicable.

## Abbreviations

CNS: Central nervous system
WHO: World Health Organization
ETMR: Embryonal tumor with multilayered rosettes
ENIGMA: The Evidence-based Network for the Interpretation of Germline Mutant Alleles
